# Time-dependent changes of plasma inflammatory biomarkers in type A aortic dissection patients without optimal medical management

**DOI:** 10.1186/s13019-014-0199-0

**Published:** 2015-01-16

**Authors:** Jun Gu, Jia Hu, Hong-wei Zhang, Zheng-hua Xiao, Zhi Fang, Hong Qian, Ming-hua Zhong, Ying-qiang Guo, Er-yong Zhang, Ying-kang Shi, Wei Meng

**Affiliations:** Department of Cardiovascular Surgery, West China Hospital, Sichuan University, Guoxue Alley 37, Cheng du, 610041 Sichuan People’s Republic of China

**Keywords:** Type A aortic dissection, Inflammatory mediator, Interleukin-6, C-reactive protein, Tumor necrosis factor-α, Complication

## Abstract

**Objectives:**

To investigate the time-dependent changes in plasma levels of interleukin-6, C-reactive protein, and tumor necrosis factor-α in patients with type A aortic dissection (TAAD) who received unoptimal medical management since the onset of dissections.

**Design and methods:**

Plasma levels of interleukin-6, C-reactive protein, and tumor necrosis factor-α were detected by ELISA and immuno-turbidimetric assay in 92 TAAD patients at hospital admission. Blood samples from 78 patients with uncontrolled hypertension and 82 healthy volunteers were also analyzed as controls. The occurrence of TAAD-related complication and its relationship with the plasma levels of these inflammatory biomarkers was also investigated.

**Results:**

The concentrations of inflammatory mediators were significant higher in TAAD than those in the uncontrolled hypertension and the healthy group. The time to peak plasma level of IL-6.and TNF-α was shorter than that of CRP in TAAD group. In the TAAD group, 51 patients suffered TAAD-related complications, and their plasma level of CRP was significantly higher than that in patients without TAAD-related complications (94.5 ± 58.8 mg/L versus 47.4 ± 47.8 mg/L, p < 0.001). Also, CRP levels strongly correlated with the value of PaO_2_/FiO_2_ ratio (r = −0.69, p < 0.001) and creatinine (r = 0.60, p < 0.001). The time to the peak level of CRP was shorter and the duration of persistently high CRP level was longer in the complication group than those in the complication-free group.

**Conclusions:**

Elevated and persistently high levels of plasma CRP, IL-6 and TNF-α were associated with progressively development of the TAAD. The changing pattern of CRP might be a marker for diagnosis and prophylactic treatment of complications. Our findings suggested a critical role of the inflammation in the progression of dissection and TAAD-related complications.

## Background

The Stanford type A aortic dissection (TAAD) is a catastrophic cardiovascular condition associated with severe morbidity and mortality [1,2]. Patients without optimal medical treatment have a mortality rate of 50-68% during the first 48 hours after an acute event, with a mean mortality of up to 1% per hour, and reaches as high as 90% within 3 months [3]. Despite the continuous advances of diagnostic technologies during recent years [4], it remains difficult to assess the progression of TAAD accurately.

Aortic dissection is characterized by medial degeneration with intimal tear and crossing of blood into the artery wall, leading to the formation of a false lumen and subsequent systemic inflammatory responses [5]. Interleukin-6 (IL-6), C-reactive protein (CRP), and tumor necrosis factor-α (TNF-α) are major pro-inflammatory cytokines, and their increasing levels have been demonstrated to be closely related to the progression of dissection and TAAD-related complications [6,7]. However, the expression of these inflammatory biomarkers might be significantly affected by several anti-hypertensive and anti-inflammatory medications, and thus their clinical value might be greatly compromised [8]. Therefore, the present study was designed to investigate the plasma levels of IL-6, CRP, and TNF-α in TAAD patients who admitted without optimal medical management since the onset of symptoms. In addition, the potential relationship between the expression levels of these inflammatory biomarkers and TAAD-related complications was also determined.

## Methods

### Patient profile

The present study was a prospective, single-center, observational study. From August 2013 to February 2014, 156 patients admitted with TAAD at West China hospital were screened. Of these patients, 138 patients (88.5%) were transferred from rural and community hospitals and patients there received unoptimal medical management. After admission, all patients were managed according to current guidelines and 118 patients (75.6%) were treated surgically. Patients received stringent anti-hypertensive management, or treatment with statins, angiotensin-converting enzyme inhibitors or angiotensin receptor blocker within last 3 months at the time of enrollment were excluded. Moreover, patients with history of autoimmune or inflammatory systemic disease were also excluded. Of the 156 patients screened for participation, 21 patients refused participation in the study, 43 patients met at least one exclusion criteria, leaving 92 patients in the study (TAAD group). Meanwhile, 78 patients with uncontrolled hypertension alone (uHT group) and 82 healthy volunteers (Control group) selected among those attending our outpatient department were also enrolled in the present study. Our investigation was approved by the Institutional Review Board of West China Hospital in compliance with the Declaration of Helsinki (reference number: 2012150). Written informed consent was obtained from all patients before inclusion in this study.

### Definitions and experimental protocol

**Figure 1:**
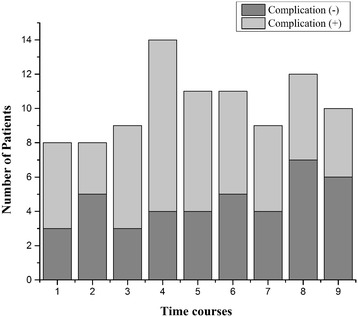
**The distribution of patients with or without TAAD-related complications.**

The diagnosis of TAAD was confirmed in all patients according to the Stanford classification. Patients were divided according to the time of admission since the onset of symptoms (<0.5 day for T1, 0.5-1 day for T2, 1–2 days for T3, 2–4 days for T4, 4–7 days for T5, 7–14 days for T6, 14–30 days for T7, 30–60 days for T8, > 60 days for T9) (Figure 1). The dissection was considered as acute if the time from the onset of the symptoms to admission was within 14 days, while chronic TAAD is that over 14 days. The uncontrolled hypertension was diagnosed by a clinic record of systolic blood pressure ≥ 140 mmHg, and/or diastolic blood pressure ≥ 90 mmHg under the antihypertensive treatment. Serum creatinine in patients without a history of renal dysfunction exceeded 150% of the normal high limit was considered to be TAAD-related renal injury [9]. PaO_2_/FiO_2_ ratio ≤ 300 was considered to be lung injury [10]. Other specifications of TAAD-related complication were defined according to 2014 European Society of Cardiology guidelines on the diagnosis and treatment of aortic diseases [4].

### Assessment of plasma inflammatory biomarkers

Heparin-anticoagulant venous blood was drawn at admission from all the patients. The plasma was obtained after centrifuging the blood at 1000 g for 15 min at 4°C and then stored at −80°C for further analysis. The concentrations of plasma TNF-α and IL-6 were measured and calculated by using ELISA technique (R&D Systems, Minneapolis, MN, USA) according to the manufacturer’s instructions. The plasma level of CRP was determined by immuno-turbidimetric assay (Beckman Assay 360, Bera, CA, USA). The detection limits were 0.5 pg/ml, 0.7 pg/ml and 0.17 mg/L for TNF -α, IL-6 and CRP, respectively.

#### Statistical analysis

Continuous variables are expressed as mean ± standard deviation, and categorical data as percentages or absolute numbers unless otherwise specified. Independent continuous variables were compared by unpaired Student’s *t* test for normally distributed data, and Mann–Whitney U-test was used for the comparison of parameters that did not exhibit a normal distribution. Categorical variables were compared using Chi-square test or Fisher’s exact test or other nonparametric tests as appropriate. The relationship between the values of inflammatory biomarkers and the incidence of TAAD-related complications was assessed by Spearman’s correlation. Two-tailed p value less than 0.05 was considered statistically significant. Statistical analyses were performed using SPSS version 16.0 (SPSS, Inc, Chicago, IL).

## Results

### Demographics of patients

Apart from the plasma levels of inflammatory cytokines at admission, no significant differences were observed regarding age, sex and other clinical variables between the TAAD group and the uHT group. In addition, patients in the TAAD and uHT groups tended to be older than healthy volunteers. Baseline data of the TAAD group and other 2 control groups are summarized in Table 1.

**Table 1:** **Baseline clinical characteristics of patients**

**Variables**	**Aortic dissection n = 92**	**Hypertension n = 78**	**Healthy controls n = 82**
Age (year)	53.2 ± 11.2	55.8 ± 13.6	50.4 ± 10.2
Males, n (%)	69 (75.0)	52 (66.7)	56 (68.3)
Smoking history, n (%)	50 (54.3)	36 (46.2)	39 (47.6)
Hypertension, n (%)	75 (81.5)	78 (100)	-
SBP, mmHg	165 ± 19^*^	158 ± 21^*^	112 ± 23
DBP, mmHg	98 ± 15^*^	88 ± 16^*^	74 ± 17
Medications at admission			
Beta receptor blocker, n (%)	17 (18.5)	19 (24.4)	-
Calcium channel bloker, n (%)	21 (22.8)	18 (23.1)	-
Alpha receptor blocker, n (%)	9 (9.8)	5 (6.4)	-
Total cholesterol (mmol/l)	5.37 ± 1.22	5.35 ± 0.98	5.03 ± 1.24
Triglyceride (mmol/l)	1.42 ± 1.31	1.57 ± 1.34	1.43 ± 1.12
Hemoglobin (g/L)	119.3 ± 23.4	124.7 ± 21.8	128.5 ± 25.6
Plasma inflammatory mediators			
IL-6 (pg/ml)	50.41 ± 42.95^*§^	5.82 ± 2.49	4.42 ± 2.12
CRP (mg/L)	68.02 ± 35.75^*§^	4.68 ± 3.89	3.08 ± 2.46
TNF-α (pg/ml)	47.52 ± 28.46^*§^	3.25 ± 1.06	2.37 ± 1.45

SBP, Systolic blood pressure; DBP, Diastolic blood pressure; IL-6, Interleukin-6; CRP, C-reactive protein; TNF-α, Tumor necrosis factor-α; Values are mean ± SD; *P < 0.05 vs healthy group, ^§^ *P* < 0.05 vs hypertension group.

### Plasma levels of inflammatory cytokines at admission

**Figure 2:**
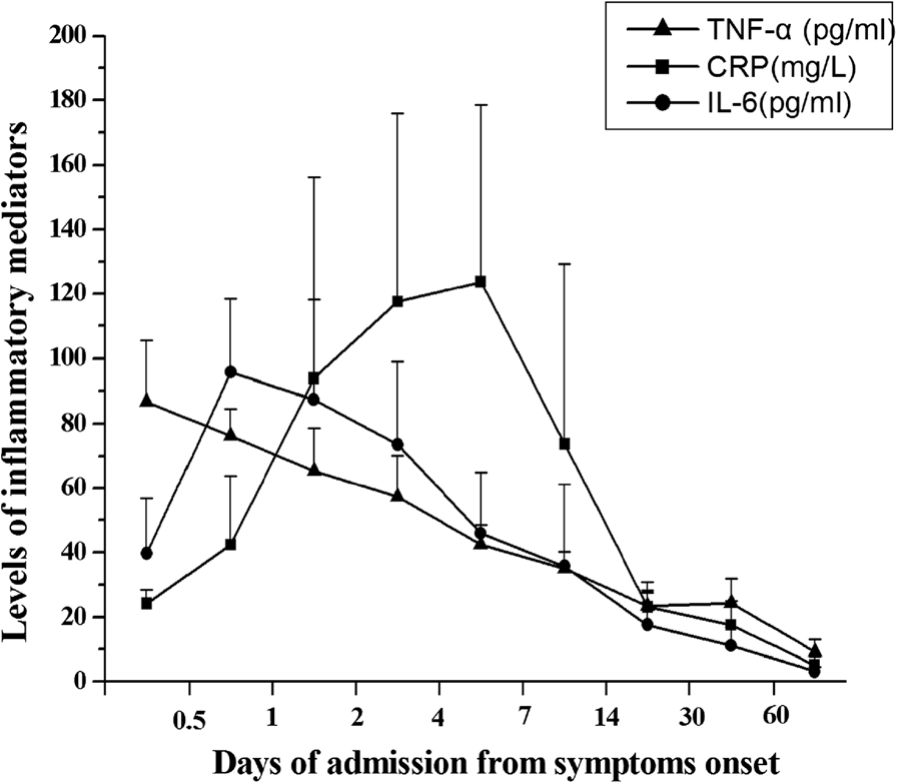
**Plasma levels of IL-6, CRP and TNF-α in different time course.**

The concentration of IL-6 was significantly higher in patients with TAAD than that in the uHT (50.41 ± 42.95 pg/ml vs. 5.82 ± 2.49 pg/ml, p < 0.05) and Control group (50.41 ± 42.95 pg/ml vs. 4.42 ± 2.12 pg/ml, p < 0.05). The level of CRP was significantly elevated in TAAD patients (68.02 ± 35.75 mg/L) compared with those in the uHT (4.68 ± 3.89 mg/L) and Control groups (3.08 ± 2.46 mg/L). Similarly, a significant increase of the plasma level of TNF-α was detected in TAAD patients (Table 1). The time to peak plasma level of IL-6.and TNF-α was shorter than that of CRP (Figure 2).

### The occurrence of TAAD-related complications

Of the 92 TAAD patients recruited in our study (Table 2), 51 patients (55.4%) suffered complications, and the distribution of patients admitted at different time points after symptoms onset was shown in Figure 1. Patients with TAAD-related complications were slightly older than patients in the no-complications cohort. Plasma CRP in the complication group was significantly higher than that in the complication-free group (94.5 ± 58.8 mg/L versus 47.4 ± 47.8 mg/L, p < 0.001). Of the 51 patients evaluated with 88 complications, the most common complications were renal dysfunction, lung injury and aortic regurgitation (as shown in Table 3).

**Table 2:** **Demographics of dissection patients with or without complications**

**Variable**	**Complication (+) group (n = 51)**	**Complication (−) group (n = 41)**	**P value**
Age(years)	61.8 ± 14.4	59.9 ± 13.8	0.458
Male, n (%)	40 (78.4)	29 (70.7)	0.244
Admission			
SBP, mmHg	142.5 ± 27.5	138.4 ± 32.7	0.211
DBP, mmHg	76.5 ± 15.8	74.3 ± 17.5	0.583
Hemoglobin (g/L)	128.4 ± 24.5	125.3 ± 19.8	0.634
Total cholesterol (mg/dl)	170.4 ± 37.8	154.7 ± 24.5	0.187
Triglyceride (mmol/L)	1.53 ± 1.05	1.46 ± 0.98	0.359
CRP (mg/L)	83.2 ± 60.7	41.9 ± 41.3	**<0.001**

SBP, Systolic blood pressure; DBP, Diastolic blood pressure.

**Table 3:** **Distribution of TAAD-related complications in the complication (+) group**

	**Complications, n (%)**
Total Complications	88 (100.0)
Aortic regurgitation	13 (14.8)
Myocardial ischemia/infarction	7 (8.0)
Congestive heart failure	6 (6.8)
Large pleural effusions	6 (6.8)
Syncope	7 (8.0)
Neurological symptoms	8 (9.1)
Mesenteric ischemia	4 (4.5)
Renal dysfunction	22 (25.0)
Lung injury	15 (17.0)

### The values of inflammatory biomarkers in relation to complications

**Figure 3:**
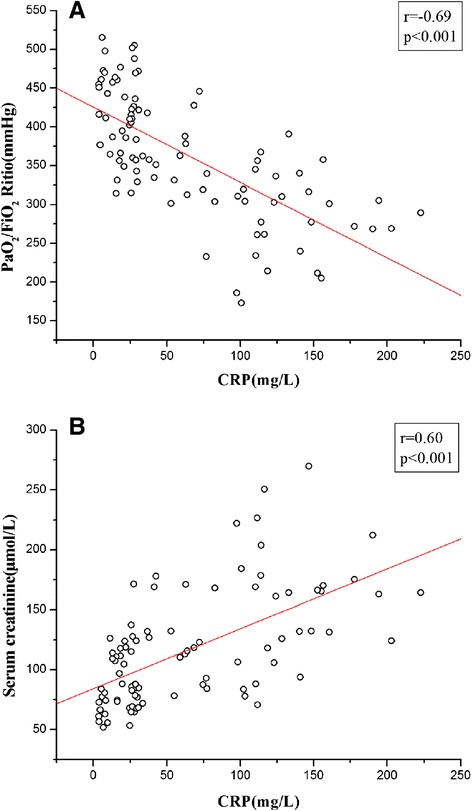
**The correlations between CRP and PaO2/FiO2 ratio (A) and serum creatinine (B).**

**Figure 4:**
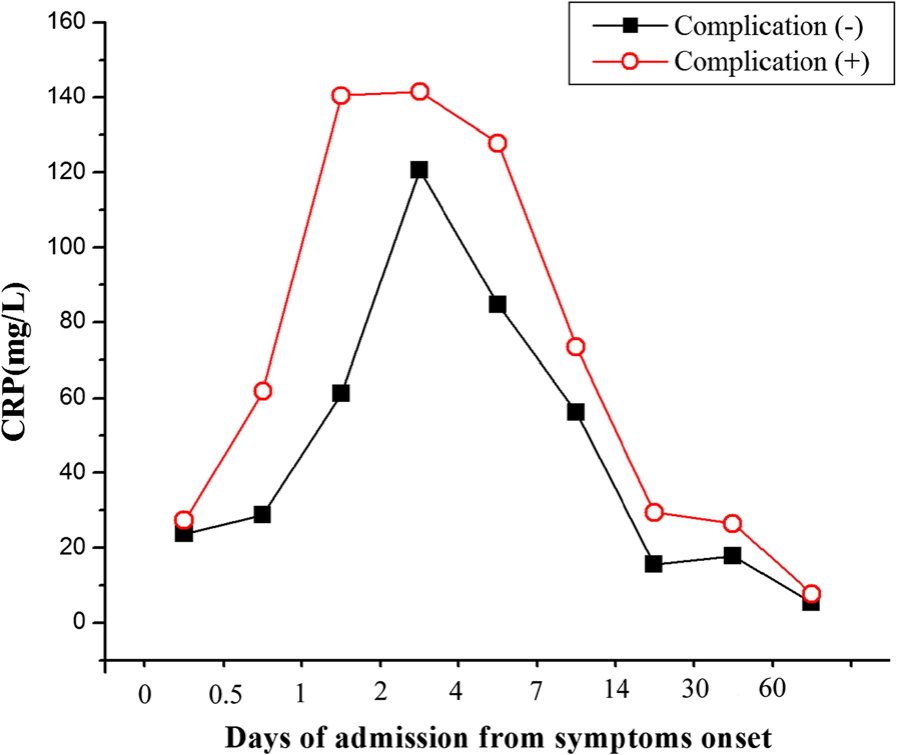
**The plasma levels of CRP in the complication (+) group and complication (−) group.** The time to peak level of CRP was shorter and the duration of persistently high CRP level was longer in the complication (+) group than those in the complication (−) group.

In TAAD patients, a significant negative correlation was found between CRP expression and PaO2/FiO2 ratio (r = −0.69, P < 0.001) (Figure 3A), whereas the relationship between the plasma level of CRP and serum creatinine demonstrated a negative correlation (r = −0.60, P < 0.001) (Figure 3B). The levels of TNF-α and IL-6 were not significantly correlated with the value of PaO_2_/FiO_2_ ratio and serum creatinine. The time-dependent changes of CRP level in the complicationgroup (51 cases) and complication-free group (41 cases) were shown in Figure 4. In the complication (−) group, the time to peak CRP (average 120.5 mg/L) was in T4 and then declined immediately. In the complication group, the CRP pattern reached to peak (average 140.5 mg/L) in T3 when earlier than complication-free group. Of note, there was a wider range time (T3-T5) of peak CRP in the complication group than that in the complication-free group.

## Discussion

In the present study, we showed that the plasma levels of CRP, IL-6 and TNF-α were significantly increased in acute TAAD patients (before T6) compared to those in uncontrolled hypertension and healthy volunteers. The expression levels of these inflammatory cytokines peaked at the acute phase of TAAD and gradually declined at different stages of TAAD progression. More importantly, the time-dependent changes of CRP level, particularly the CRP pattern provided more information in terms of prediction of TAAD-related inflammatory complications. Our data strongly suggested a critical role of inflammatory responses in the development of TAAD and resultant systemic complications.

CRP, a circulating and nonspecific inflammatory biomarker, has been shown to be elevated and varied with different stages of aortic dissection [7,11-13]. Peak plasma level of CRP at admission is regarded as a predictor of adverse short- and long-term events in patients with TAAD [7]. Consistent with previous studies, we demonstrated a time-dependent change of CRP, which peaked in patients admitted on the 4^th^ day (T5) after symptoms onset and declined thereafter. Secondly, because CRP is a non-specific inflammatory marker, it reflects not only the TAAD itself but also systemic inflammatory responses, leadig to pulmonary or renal injury [14,15]. More importantly, in the complication group, the time to peak CRP was earlier and the duration of peak CRP was longer than in the complication-free group. These findings indicated a contributory role of inflammation in the progression of dissection, and further suggested that TAAD patients with persistently high CRP levels during the first 7 days from symptoms onset might be of great risk for major adverse events.

Significant elevation of IL-6 and TNF-α levels were also detected in patients admitted at the acute phase of TAAD (before T7) when compared with other 2 control groups. However, in patients with chronic TAAD (T7-T9), no significant difference between the chronic TAAD subgroup and the uHT group was observed. In addition, the changing patterns of these two inflammatory cytokines were slightly different. Nevertheless, the plasma levels of IL-6 and TNF-α both peaked during the 12 hrs to 24 hrs period, which was much earlier than CRP. Current evidence demonstrated a significant infiltration of macrophages and neutrophils in the dissected aortic wall [16]. These recruited leukocytes could release a series of pro-inflammatory cytokines, which would further accelerate the progression of dissection and lead to systemic complications. Since CRP is produced mainly in liver by the stimulation of many inflammatory cytokines [17], it is understandable that the plasma levels of leukocytes-derived cytokines like IL-6.and TNF-α peaked earlier than CRP.

In the current study, the average values of CRP, IL-6 and TNF-α in patients with chronic TAAD and hypertension tended to be higher than those in the healthy controls (Chronic TAAD vs. Healthy, P = 0.06; uHT vs. Healthy, P = 0.09). As the mechanism of TAAD remained large unknown [18], our findings accordingly confirmed that inflammation was closely involved into the pathogenesis of hypertension-induced aortic wall injury and the development of aortic dissection. Thus it is also important to follow up the plasma levels of inflammatory cytokines in patients with uncontrolled hypertension. If these biomarkers do not decrease after stringent control of blood pressure, there could be a potential risk of aneurysmal dilatation of the aorta that might lead to future dissection, and therefore, additional anti-inflammatory medications like statins for such patients might be a reasonable choice.

### Limitation

There are several limitations in the present study. Firstly, because of the absence of serial blood samples spanning the first few hours period of TAAD progression, it is difficult for us to fully elucidate the changing patterns of CRP, IL-6.and TNF-α and their clinical values for diagnosis and prophylactic treatment of complications. Secondly, in order to avoid significant influences of several known drugs on the expression levels of inflammatory biomarkers, we recruited TAAD patients without optimal medical management. However, the rationality of the inclusion criteria remains to be fully discussed. Nevertheless, to the best of our knowledge, the present study firstly provided information on the changes of the inflammatory biomarkers, which were less affected by anti-hypertensive and anti-inflammatory medications. In addition, we observed such a surprisingly high percentage of inappropriately treated patients from rural areas (78/92, 84.7%), indicating the urgent necessity and importance of the primary prevention for hypertension-related disease in the rural areas of China. Although this study was strengthened by its prospective design, the patient population is relatively small, and we cannot exclude the possibility that other confounders such as genetic factors (Marfan syndrome etc.), smoking, diabetes and use of β-blockers and missed medication history may have influenced our results.

## Conclusions

In this small sample size, prospective study, we demonstrated that increased plasma CRP, IL-6 and TNF-α were significantly associated with TAAD. More importantly, the changing pattern of CRP might be a marker for diagnosis and prophylactic treatment of complications. Taken together, our data clearly indicated the critical role of inflammation in the progression of dissection and TAAD-related complications.

### Patient informed consent

Written informed consent was obtained from the patients for publication of this study. Copies of the written consent are available for review by the Editor-in-Chief of this journal.

## Abbreviations

TAAD: Type A aortic dissection
IL-6: Interleukin-6
CRP: C-reactive protein
TNF-α: Tumor necrosis factor-α
